# Molecular mapping of QTL alleles of *Brassica oleracea* affecting days to flowering and photosensitivity in spring *Brassica napus*

**DOI:** 10.1371/journal.pone.0189723

**Published:** 2018-01-10

**Authors:** Habibur Rahman, Rick A. Bennett, Berisso Kebede

**Affiliations:** Department of Agricultural, Food and Nutritional Science, University of Alberta, Edmonton, Canada; University of Guelph, CANADA

## Abstract

Earliness of flowering and maturity are important traits in spring *Brassica napus* canola–whether grown under long- or short-day condition. By use of a spring *B*. *napus* mapping population carrying the genome content of *B*. *oleracea* and testing this population under 10 to 18 h photoperiod and 18 to 20 ^0^C (day) temperature conditions, we identified a major QTL on the chromosome C1 affecting flowering time without being influenced by photoperiod and temperature, and a major QTL on C9 affecting flowering time under a short photoperiod (10 h); in both cases, the QTL alleles reducing the number of days to flowering in *B*. *napus* were introgressed from the late flowering species *B*. *oleracea*. Additive effect of the C1 QTL allele at 14 to18 h photoperiod was 1.1 to 2.9 days; however, the same QTL allele exerted an additive effect of 6.2 days at 10 h photoperiod. Additive effect of the C9 QTL at 10 h photoperiod was 2.8 days. These two QTL also showed significant interaction in the control of flowering only under a short-day (10 h photoperiod) condition with an effect of 2.3 days. A few additional QTL were also detected on the chromosomes C2 and C8; however, none of these QTL could be detected under all photoperiod and temperature conditions. BLASTn search identified several putative flowering time genes on the chromosomes C1 and C9 and located the physical position of the QTL markers in the Brassica genome; however, only a few of these genes were found within the QTL region. Thus, the molecular markers and the genomic regions identified in this research could potentially be used in breeding for the development of early flowering photoinsensitive *B*. *napus* canola cultivars, as well as for identification of candidate genes involved in flowering time variation and photosensitivity.

## Introduction

Early flowering and maturity are important traits for spring oilseed *B*. *napus* for growing this crop in temperate regions, under long-day conditions, as well as in sub-tropical or tropical regions, where the crop is grown during winter under a shorter day [1, 2]. In North America and Europe, spring canola requires about 4–5 months from seeding to harvest, while in a sub-tropical zone, such as Australia, it may require up to 6–7 months.

According to Habekotté [3] an oilseed *B*. *napus* cultivar possessing the properties of early flowering and delayed maturity and high leaf area index is expected to produce high seed yield. The beneficial effect of early flowering and delayed maturity apparently result from longer post-flowering period. Gan *et al*. [4] found a positive association of the duration of post-flowering period and seed yield in spring *B*. *napus* canola grown in western Canada. In temperate regions like western Canada, spring canola is grown in the summer where the air temperature in mid-summer can reach up to 30°C or even higher. Gan *et al*. [5] found a significant negative effect of high temperature stress at bud formation to silique development stage on seed yield in spring *B*. *napus* canola. Angadi *et al*. [6] also found similar effect of high temperature in a growth chamber experiment. The high heat stress at the reproductive stage of the crop can be avoided to some extent by seeding the crop early in the season or delaying the seeding. However, early seeding can delay seed germination due to low soil temperature [7], as well as can subject the seedlings to a damage caused by spring-frost and this can delay flowering and maturity, while late seeding can delay maturity and this can subject the crop to a damage caused by fall-frost. Frost damage, whether it is in early or late growth stage, reduces seed yield; frost damage in the late growth stage also increases chlorophyll content in the seed and thereby adversely affect seed quality [8–10].

In tropical or sub-tropical regions, Brassica oilseed crops are grown as winter crops. In these regions, high temperature during terminal stage of the crop result significant yield loss; early flowering and maturing cultivars generally performs better there [11]. Earliness of flowering is also an important trait for winter/semi-winter or vernalization-responsive *B*. *napus*–this trait shows a positive association with seed yield [12, 13]. Days to flowering correlates well with days to maturity in spring (*r* = 0.74**) and winter (*r* = 0.38**) *B*. *napus* [14, 15] as well as in *B*. *juncea* [16]; therefore, this trait is an important objective of breeding early maturing *B*. *napus* cultivars.

*Brassica napus* is considered a long-day plant [1, 17], the long days shorten the duration from seeding to flowering. According to King and Kondra [18], the minimum optimum photoperiod for the Canadian spring *B*. *napus* is about 18 hrs. The day length in the major Canadian canola growing regions is about 15 to 17 h prior to flowering; this indicates that the maximum optimum photoperiod may not be reached under this growth condition. Therefore, identification of the gene(s) controlling photoinsensitivity or flowering under a short-day condition will benefit breeders for designing a knowledge-based program for the development of an early flowering/maturing and high yielding *B*. *napus* cultivar. Very little information is available on mapping of the photosensitive genes affecting flowering time under a short day condition. QTL mapping studies so far identified the chromosomes A2, A3, A10, C3, C4, C5 and C9 carry photosensitivity genes [19–22]. According to Robertson *et al*. [1], the late flowering spring *B*. *napus* show a greater response to vernalization and photoperiod than the early flowering *B*. *napus*, i.e. early flowering types are less sensitive to photoperiod. Therefore, development of an early flowering spring *B*. *napus* cultivar will be important not only for the temperate regions, but also for the geographical regions where this crop is grown in winter under a short photoperiod condition.

The objective of this research was to understand the genetic control of earliness of flowering and photosensitivity in the C genome of *B*. *napus* carrying the genome content introgressed from *B*. *oleracea*.

## Materials and methods

### Plant materials

A spring *B*. *napus* doubled haploid (DH) population of 95 lines derived from an F_1_ of Hi-Q × RIL-144 cross was used in this study. Hi-Q is a spring *B*. *napus* canola cultivar, developed by the University of Alberta, while RIL-144 is an early flowering *B*. *napus* line (F_6_), developed from *B*. *napus* × *B*. *oleracea* var. *alboglabra* (CC, 2*n* = 18) interspecific cross with selection for earliness of flowering [23]. This line flowers about a week earlier than most of the *B*. *napus* cultivars and breeding lines including Hi-Q.

### Evaluation for days to flowering

The DH lines were grown in growth chambers under the following photoperiod and temperature conditions for evaluation of days to flowering:

1. 10 h photoperiod and 18 ^0^C constant temperature,
2. 14 h photoperiod and 18 ^0^C constant temperature,
3. 18 h photoperiod and 18 ^0^C constant temperature,
4. 16 h photoperiod and 18/8 ^0^C (day/night) temperature,
5. 16 h photoperiod and 20 ^0^C constant temperature,

Photosynthetic flux density of 450 μE (mV) m^2^ s^1^ at plant level and relative humidity of 50% was maintained in the growth chambers.

The experiments *i* to *iii* were carried out primarily to identify the QTL associated with photoperiod-responsive flowering; experiment *iv* represents the photoperiod and temperature conditions in Alberta, Canada before canola starts to flower (May–June) based on 30 years average of high and low temperature (http://www.theweathernetwork.com/statistics/CL3012209/caab0103/); the experiment *v* was included to represent an increase in day and night temperature compared to the experiment *iv*. The growth chamber experiments of *i*, *ii*, *iii*, *iv* and *v* were repeated 2, 3, 3, 3 and 5 times, respectively, and these constituted the number of replications. Number plants grown in each replication of the experiments *i*, *ii* and *iii* was three, and in case of the experiments *iv* and *v*, it was two; the average value of these plants was used for statistical analysis. The two parents and their F_1_, as well as an early flowering *B*. *napus* cv. Peace, were included in the experiments for comparison. Peace is one of the most early flowering *B*. *napus* cultivar available in the Canola Program of the University of Alberta; this cultivar was registered in Canada in 2001. For all these experiments, except the 16 h photoperiod 18/8°C experiment, the plants were grown in 32-cell tray with pot size of 7 cm × 7 cm × 9 cm (L × W × D) filled with Sunshine Professional Growing Mix (Sunshine Horticulture, 15831 N.E. Bellevue, USA). In case of the 16 h photoperiod 18/8°C experiment, plants were grown in 10 cm × 10 cm × 10 cm (L × W × D) size pots. Data on days to flowering was recorded at three-open-flower stage.

### Molecular mapping of days to flowering

Simple sequence repeat (SSR) markers collected from different sources, such as Agriculture and Agri-Food Canada (AAFC) (http://aafc-aac.usask.ca/BrassicaMAST/) and Brassica Genome Gateway (http://brassica.nbi.ac.uk/), and markers published by different researchers [24–27], as well as markers developed based on *Brassica* homologous sequences of the flowering time genes of *A*. *thaliana* [28] (https://www.arabidopsis.org/)) and flowering time genes of *B*. *oleracea* and *B*. *rapa* obtained from NCBI (The National Center for Biotechnology Information, http://www.ncbi.nlm.nih.gov/) database, *Brassica* flowering time gene-based markers published by Razi *et al*. [29] and Wang *et al*. [30], and markers obtained from Professor Christian Jung, University of Kiel, Germany, and AFLP markers were used for construction of a genetic linkage map and QTL mapping. The detail of these markers, genotyping the population, and construction of the genetic linkage map is described elsewhere [31]. QTL analysis was done using the software QTL cartographer ver. 2.5 (http://statgen.ncsu.edu/qtlcart/WQTLCart.htm) [32] and following single marker analysis and composite interval mapping (CIM) methods. In case of CIM method, the likelihood of a QTL and its effect was estimated at every 1 cM interval in a window of 10 cM. To declare a QTL, permutation tests were done (*n* = 1000 permutations) to determine the threshold for LOD or likelihood-ratio scores; an auto cofactor (type I error rate) of *P* = 0.05 was used. A 99% confidence interval was used to determine the QTL position. The additive effect of the QTL and the proportion of the total phenotypic variance (R^2^) explained by the QTL was obtained from the QTL peak area. To confirm the QTL, the whole procedure was repeated at least four times. Digenic epistasis analysis for additive × additive genes and epistasis × environment interaction was done using QTLNetwork 2.0 (http://ibi.zju.edu.cn/software/qtlnetwork/download.htm) [33].

BLASTn search was conducted using primer sequences of the QTL markers in the genome sequence assembly of *B*. *napus* (gene bank database Bra_napus_v2.0) maintained in NCBI to locate the physical position of the QTL as well as to identify putative genes in these genomic regions.

### Statistical analysis

Analysis of variance (ANOVA) with data from the 10, 14 and 18 h photoperiod (18°C constant temperature) experiments was conducted using PROC MIXED procedure of SAS [34] where the effect of genotype was nested in different photoperiods (10h, 14h and 18h) using the following model:

Yij=μ+Gi+Pj+G(P)ij+eij,

where, Y*ij* is observation of genotype *i* in environment (photoperiod) *j*, μ is the general grand mean, G*i* is genotype *i* in photoperiod (either 10h, 14h or 18h) *j*, and G(P)*ij* is the nested effect of genotype *i* in photoperiod *j*, and e*ij* is the residual error of genotype *i* in photoperiod *j*.

Descriptive statistics, such as mean, standard deviation and variances, as well as Spearman’s correlation coefficients were calculated using the SAS software program of version 9.3 [34].

## Results

### Evaluation of the DH lines

In all the experiments, conducted at different photoperiod and temperature conditions, the parent RIL-144 flowered earlier than Hi-Q. The variance analysis showed significant difference existed among the DH lines and the photoperiod had profound influence on days to flowering. The genotype × photoperiod interaction was also significant (Table A in S1 File) for this trait.

Coefficient of correlation between the replications was calculated for each of the experiments for days to flowering. In all cases, significant correlation was found between the replications (Table B in S1 File); this indicates that the DH lines behaved similarly for days to flowering under the same growth condition; therefore, data from the replications was pooled and LSmeans values were used for QTL mapping.

**Fig 1:**
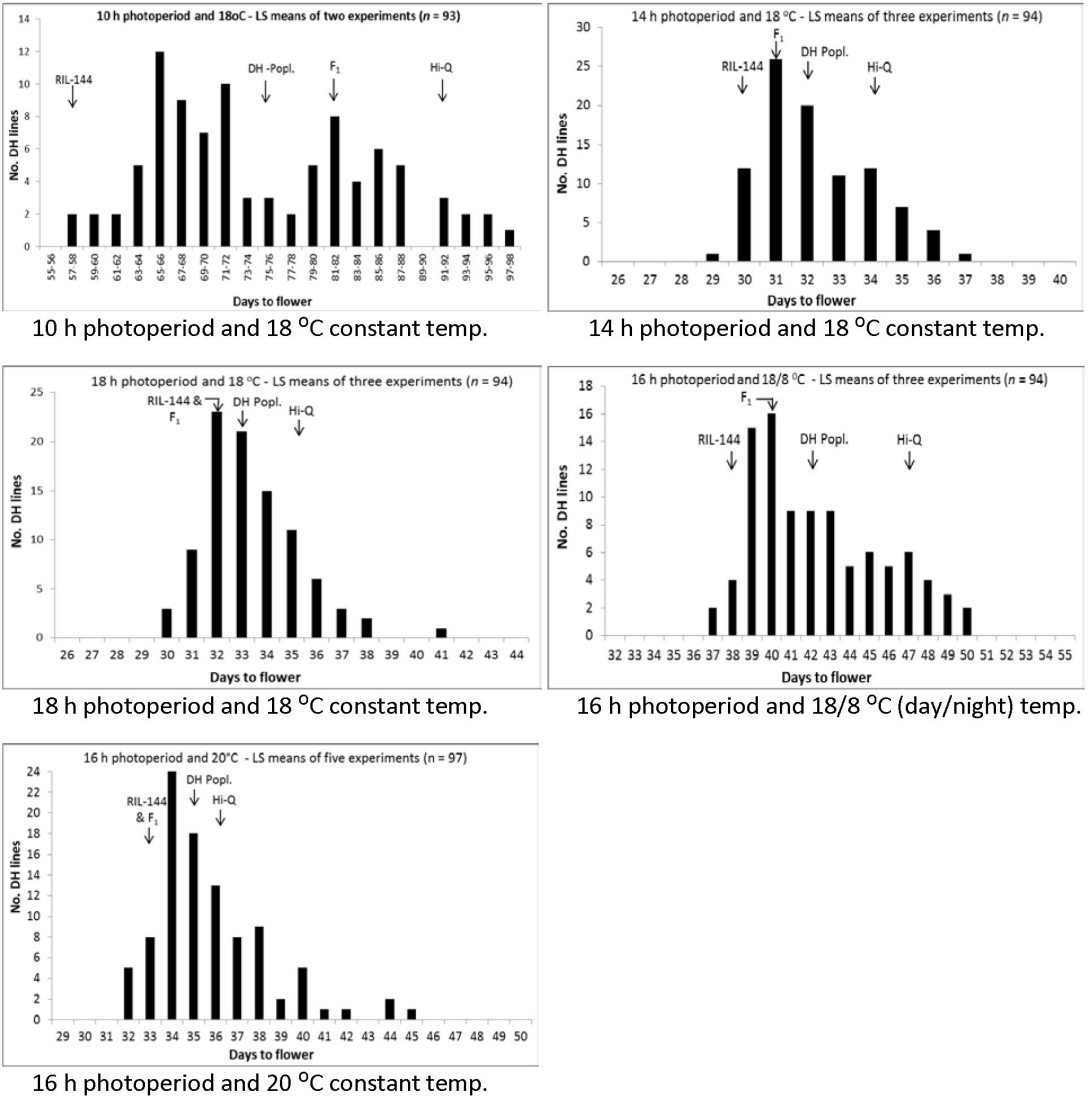
Frequency distributions of the DH lines of Hi-Q × RIL-144 cross of *Brassica napus*, grown under different photoperiod and temperature in growth chamber, for days to flowering.

Of the three photoperiod experiments conducted at 18 ^0^C constant temperature, the DH population grown at 10 h photoperiod took significantly longer time (> 40 days) to flower compared to 14 or 18 h photoperiod; however, the difference between the 14 and 18 h experiment was not statistically significant (*p* = 0.0374) (Table 1). At a 10 h photoperiod condition, the early flowering parent RIL-144 required 57 days to flower while Hi-Q required 92 days to flower; in contrast, the difference between these two parents to flower at 14 and 18 h photoperiod was only 3–4 days (Fig 1). This indicates that short photoperiod had stronger influence on the parent Hi-Q and this also reflected in the DH population (Fig 1). The distribution of the DH population grown at 10 h photoperiod was almost bimodal, while a continuous variation was found when the population was grown under all other day length and temperature conditions (Fig 1).

**Table 1:** Least squares mean difference for days to flowering of the DH population of Hi-Q × RIL-144 cross of *Brassica napus* grown in growth chamber under three different photoperiod and constant temperature of 18 ^0^C.

Photoperiod I	Photoperiod II	LSmeans difference	Standard error	*t*-value	*P*	Adjusted *P (*Dunnett’s test)
10h	14h	42.7	0.6	75.34	< .0001	< .0001
10h	18h	41.6	0.6	73.40	< .0001	< .0001
14h	18h	-1.1	0.5	-2.09	0.0374	0.1006

At 14 to 18 h photoperiod and constant temperature of 18 or 20 ^0^C, the parents and the DH population on average required ≤36 days to flower; however, when the night temperature was reduced to 8 ^0^C, the parents and the DH population took significantly longer time to flower. Thus, an effect of temperature was also evident in this population. This was also evident from the shorter time required to flower under a 16 h photoperiod and 20 ^0^C temperature condition as compared to the time required to flower under the same photoperiod but 18/8 ^0^C (day/night) temperature condition (Table C in S1 File)

### Correlation between the growth chamber experiments for days to flowering

In general, coefficient of correlation between the 14, 16 and 18 h experiments (*r* = 0.607–0.744, mean 0.656) were greater than the coefficient of correlation of the 10 h experiment with the 14, 16 and 18 h experiments (*r* = 0.475–0.578, mean 0.524) (Table D in S1 File). This might be due to the involvement of additional genetic factor in the control of days to flowering under a short day (10 h) condition. Coefficient of correlation between the experiments (except the 10 h) seeded in small pot (*r* = 0.624–0.744, mean 0.680) was slightly greater than the coefficient of correlation of the experiments seeded in large vs. small pot (*r* = 0.607–0.668, mean 0.631); however, this difference was not as great as the difference between the 14 to 18 h experiments and 10 h vs. 14–18 h experiments.

### QTL mapping

**Fig 2:**
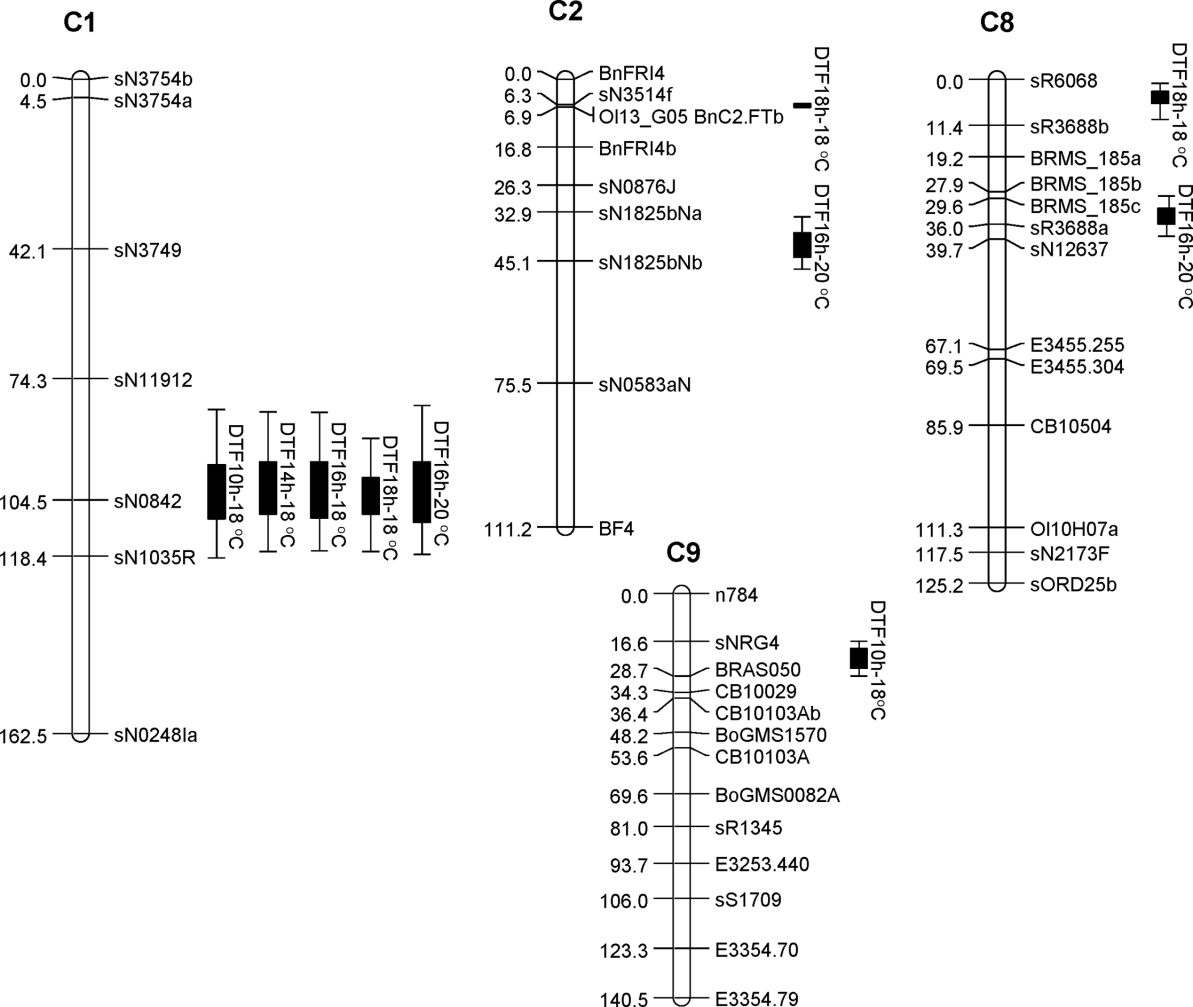
QTL mapping of days to flowering under different photoperiod and temperature conditions by use of a doubled haploid (DH) population derived from Hi-Q × RIL-144 cross of *Brassica napus* carrying the C genome content of *B*. *oleracea* var. *alboglabra*.

A total of six QTL were detected on the linkage groups C1, C2, C8 and C9 by CIM method of which four QTL of C1, C2, C8 and C9 were detected with LOD score of >2.5 while the other two QTL of C2 and C8 were detected with LOD score ≤2.5 (Table 2 and Fig 2). Among these, the QTL of C1, located between the flanking markers sN11912 and sN1035R, was detected in all experiments conducted at different photoperiod and temperature conditions. In all cases, this QTL was detected with LOD score of >3 and explained 21.7 to 59.7% of the total phenotypic variance. Additive effect of this QTL allele was one to three days in most cases except the 10 h experiment where additive effect of this QTL was 6.2 days. Single marker analysis also showed strong association (*p* = 0.000–0.001) of the marker sN0842 from this QTL region with days to flowering under all conditions (Table 3).

**Table 2:** QTL detected for days to flowering (DTF) under different photoperiod and temperature conditions by composite interval mapping by use of a doubled haploid population derived from Hi-Q × RIL144 cross of *Brassica napus* carrying the C genome content of *B*. *oleracea* var. *alboglabra*.

QTL	Linkage group	Marker interval	Confidence interval (cM)	Peak of putative QTL (cM)	R^2^ ^a^ (%)	Additive effect^b^	LOD score
q1DTF10h-18 ^o^C	C1	sN11912—sN1035R	81.0–118.9	104.2	42.2	6.2	11.9
q9DTF10h-18 ^o^C	C9	sNRG42—BRAS050	16.6–34.4	27.6	37.1	2.8	2.8
q1DTF14h-18 ^o^C	C1	sN11912—sN1035R	81.6–118.1	102.6	29.8	1.1	3.6
q2DTF14h-18 ^o^C	C2	sN3514f - BnC2.FTb	6.3–6.9	6.8	12.1	0.7	2.2
q1DTF18h-18 ^o^C	C1	sN11912—sN1035R	89.5–118.1	106.8	34.2	1.3	3.9
q8DTF18h-18 ^o^C	C8	sR6068—sR3688b	0.0–11.4	5.6	24.6	-2.3	2.5
q1DTF16h-18/8 ^o^C	C1	sN11912—sN1035R	81.6–118.1	102.6	59.7	2.9	11.9
q1DTF16h-20 ^o^C	C1	sN11912—sN1035R	78.0–118.4	101.4	21.7	2.3	4.2
q2DTF16h-20 ^o^C	C2	sN1825bNa—sN1825bNb	32.9–45.1	38.5	14.2	1.5	3.4
q8DTF16h-20 ^o^C	C8	BRMS_185c - sR3688a	29.6–36.0	32.5	11.4	-4.3	2.7

^*a*^*R*^2^ is the percentage of phenotypic variation explained by each QTL.

^*b*^Additive effect is the effect of substitution of Hi-Q allele by RIL144 allele; positive values indicates that the allele from Hi-Q delays days to flower, i.e. increases the number of days to flower.

**Table 3:** QTL detected for days to flowering (DTF) under different photoperiod and temperature conditions by single marker analysis and by use of a doubled haploid population derived from Hi-Q × RIL144 cross of *Brassica napus* carrying the C genome content of *B*. *oleracea* var. *alboglabra*.

Growth condition and chromosome	Marker ID	Position	-2ln(L0/L1)a ^a^	*p*^b^
10h photoperiod 18 ^o^C				
C1	sN0842	104.5	32.4	0.000
C2	Ol13_G05	6.9	17.1	0.000
C2	BnC2.FTb	6.9	9.8	0.002
C2	sN1825bNb	45.1	5.5	0.021
C4	BRMS-001a	0.0	6.9	0.009
C4	BRMS-001b	2.1	6.7	0.011
C8	BRMS_185b	27.9	4.8	0.031
C8	BRMS_185c	29.6	4.8	0.031
C8	BRMS_185a	19.2	4.6	0.034
C9	n784	0.0	7.6	0.007
C9	BRAS050	28.7	10.9	0.001
14h photoperiod 18 ^o^C				
C1	sN0842	104.5	31.4	0.000
C2	Ol13_G05	6.9	17.4	0.000
C2	BnC2.FTb	6.9	11.2	0.001
18h photoperiod 18 ^o^C				
C1	sN0842	104.5	12.2	0.001
C2	Ol13_G05	6.9	5.6	0.020
C8	sR3688b	11.4	7.1	0.009
16h photoperiod 18/8 ^o^C				
C1	sN0842	104.5	59.6	0.000
C2	Ol13_G05	6.9	15.5	0.000
C2	BnC2.FTb	6.9	6.9	0.010
16h photoperiod 20°C				
C1	sN0842	104.5	37.8	0.000
C2	sN3514f	6.3	7.9	0.006
C2	Ol13_G05	6.9	58.5	0.000
C2	BnC2.FTb	6.9	39.0	0.000
C2	sN1825bNb	45.1	5.2	0.025
C2	sN0583aN	75.5	5.0	0.028

^a^Likelihood Ratio Test Statistic.

^b^Significance at the 5%, 1%, 0.1% and 0.01% levels for F statistic for each marker

Among the experiments with different combinations of temperature and photoperiod, the 10 h photoperiod experiment can be considered a short-day condition. Under this condition, a QTL on C9 flanked by the markers sNRG42 and BRAS050 was detected through CIM method (Table 2 and Fig 2). Single marker analysis also detected significant association of the marker BRAS050 (*p =* 0.001) from this QTL region, thus, substantiate this finding (Table 3). This QTL exerted an additive effect of 2.8 days at 10 h photoperiod condition and explained 37% of the total phenotypic variance (Table 2).

A QTL on the linkage group C2, flanked by the markers sN1825bNa and sN1825bNb, was detected in the 16 h photoperiod experiment with 20°C constant temperature (Table 2and Fig 2). This QTL explained more than 10% of the total phenotypic variance; however, it could not be detected in the other four growth chamber experiments through CIM method. Single marker analysis also showed significant association of sN1825bNb with days to flowering in this growth chamber experiment (Table 3). In contrast, the second QTL of C2 carrying the *FT* gene [30, 31] could be detected only at 14 h photoperiod 18°C temperature condition through CIM method; however, single marker analysis detected the association of the markers Ol13_G05/BnC2.FTb in all five experiments (Table 3).

Two QTL from the linkage group C8 were identified where the allele from the early flowering line RIL-144 delayed flowering in the DH population (Table 2). This is not surprising given that some of the DH lines flowered later than the late-flowering parent Hi-Q.

Digenic epistasis analysis identified an interaction of the C1 and C9 QTL regions at 10 h photoperiod 18°C temperature condition with an additive effect of 2.3 ± 0.7 days (*p* = 0.001) (Table 4); however, no epistatic interaction between these two QTL could be detected under other photoperiod and temperature conditions.

**Table 4:** Epistasis effect estimated on days to flowering (DTF) by use of a doubled haploid population derived from Hi-Q × RIL144 cross of *Brassica napus* carrying the C genome content of *B*. *oleracea* var. *alboglabra*.

Growth condition	Marker interval I /LG	Peak of putative QTL I (cM)	Confidence interval I (cM)	Marker interval II /LG	Peak of putative QTL II (cM)	Confidence interval II (cM)	AA^a^	SE^b^	P-Value
10h 18°C	sN0842—sN1035R /1	115.5	104.5–118.8	sNRG42—BRAS050 /9	21.0	16.6–28.7	2.3	0.73	0.001

^*a*^ Additive effect of the epistasis interaction between the two QTL,

^b^ Standard error

### BLASTn search

BLASTn searches against the gene bank database of *B*. *napus* identified 4,278 and 2,098 putative genes on the chromosomes C1 and C9, respectively (Table A and Table B in S2 File), of which 18 (9 + 9) found to be related to flowering time (Table 5). Scanning of the QTL region of C1 flanked by the marker sN11912 (genetic position 74.3 cM; physical position start 21,171,155 nt and end 21,171,421 nt) and sN0842 (genetic position 104.5 cM; physical position start 41,134,210 nt and end 41,134,425 nt) (physical position of the marker sN1035R fall in between the above-mentioned two markers: start 28,985,859 and end 28,986,027 nt) in NCBI database showed the presence of 1,674 genes (scanning was done on July 21, 2017). Among these, the gene ID 106374859 encoding “*zinc finger protein CONSTANS-LIKE 12-like*”, located at physical position of 32,873,610 nt (start position) to 32,875,196 nt (end position), and the gene ID 106376922 encoding “*protein EARLY FLOWERING 3-like*”, located at physical position of 33,189,464 nt (start position) and 33,192,085 nt (end position), seems to be associated with flowering time (Table 5).

**Table 5:** List of putative flowering time genes from the C genome chromosomes C1 and C9 of *Brassica napus*.

Chro-moso-me	GeneID	Aliases	Description	Other designations	Genomic nucleotide accession version	Start position on the genomic accession	End position on the genomic accession	Exon count
C1	106374237		Flowering-promoting factor 1-like protein 1	BnaC01g07130D	NC_027767.1	2180091	2180811	1
C1	106375657		Protein FRIGIDA-like	BnaC09g27290D|FRIGIDA-like protein	NC_027767.1	6352104	6354341	3
C1	106374375		Agamous-like MADS-box protein AGL19		NC_027767.1	8619501	8623147	8
C1	106373058		Agamous-like MADS-box protein AGL19		NC_027767.1	8640130	8643461	7
C1	106375854		Zinc finger protein CONSTANS-LIKE 12-like	BnaC04g43770D	NC_027767.1	9629006	9632489	6
C1	106373210		Zinc finger protein CONSTANS-LIKE 3-like		NC_027767.1	13561707	13593662	0
C1	106374511		Polycomb group protein VERNALIZATION 2-like		NC_027767.1	14274722	14277547	12
C1	106374859		Zinc finger protein CONSTANS-LIKE 12-like		NC_027767.1	32873610	32875196	5
C1	106376922		Protein EARLY FLOWERING 3-like		NC_027767.1	33189464	33192085	6
C9	106416685		Protein UPSTREAM OF FLC-like		NC_027775.1	5181013	5182924	5
C9	106416461	FLC.C2, FLC4	MADS-box protein FLOWERING LOCUS C-like	BnaC02g00490D|flowering locus C	NC_027775.1	5189964	5193553	7
C9	106417905		Protein DOWNSTREAM OF FLC-like		NC_027775.1	5195979	5197095	2
C9	106416680		Zinc finger protein CONSTANS-LIKE 6-like		NC_027775.1	9055107	9057009	3
C9	106416760		Agamous-like MADS-box protein AGL14		NC_027775.1	9768154	9772983	16
C9	106416802		Zinc finger protein CONSTANS-LIKE 6-like		NC_027775.1	11420887	11422161	2
C9	106418081		Polycomb group protein VERNALIZATION 2-like		NC_027775.1	13811400	13814225	12
C9	106416437		Putative zinc finger protein CONSTANS-LIKE 11		NC_027775.1	15121307	15123823	5
C9	106416779		Zinc finger protein CONSTANS-LIKE 15-like		NC_027775.1	15952353	15954071	0

In the case of the C9 QTL, BLASTn search failed to identify the physical position of the QTL marker BRAS050, however, the marker sNRG42 (genetic position 16.6 cM) could be positioned between 653,462 and 653,731 nt of the *B*. *napus* genome. Expected fragment size of this marker is about 205–253 bp; the deduced fragment size, based on this search, agreed well with the expected size. Of the total 2,098 putative genes of the 23,211,758 nt long C9 chromosome, nine were found to be related to flowering time (Table 5) where four seems to be *CO*-like, three *FLC*-like, one *VERN*-like and one *AGL*-like genes; however, none of these genes could be found at around the physical position of the marker sNRG42. The closest gene to this marker was a *FLC*-like which was found to be located about 4.5 Mb away from this marker, while the closest *CO*-like gene was found to be located about 8.4 Mb away from this marker. The genomic region of the marker sNRG42 found to carry two At2g44700-like genes (*F-box/kelch-repeat protein At2g44700-like*; physical positions 650,761 to 651,798 and 657,807 to 658,847 nt) (Table B in S2 File); however, function of these genes could not be found in database.

## Discussion

The genetic control of flowering time in *B*. *napus* is complex − controlled by a large number of loci and their interactions [35]. Given the fact that *B*. *napus* has a narrow genetic base [36], allelic variation for all loci may not be present in natural population of this species and this can be a hindrance for detection of all QTL affecting flowering time by use of a traditional mapping population. In this study, by using a mapping population carrying the genome content of the C genome of *B*. *oleracea* var. *alboglabra*, we detected six QTL from four linkage groups of which one was not influenced by photoperiod and temperature and one was detected only under a short day condition. Some of the QTL or genomic regions showed more than 30 cM gap between the markers; this is primarily due to close relationship between the parents RIL-144 and Hi-Q. This close relationship was also evident from the fact that only 12% of the 795 tested SSR markers were polymorphic between these two parents.

Flowering time in a plant is regulated by the genes involved in different pathways, such as photoperiod, vernalization and autonomous (flowering independent of environmental cues), and their interactions. Details of these pathways are reviewed by different researchers [37–41]. The key regulator in the photoperiod pathway is the *CONSTANS* (*CO*) gene. This gene under a long-day condition and at the end of the light phase accumulates CO protein which activates the transcription of the *FLOWERING LOCUS T* (*FT*) gene to initiate flowering. The *FT* is a central integrator gene, receive signals from different pathways. Protein of this gene interact physically with *FLOWERING LOCUS D* (*FD*) protein in the meristem and this complex activates the floral meristem identity genes, such as *APETALA 1* (*AP1*) and *CAULIFLOWER* (*CL*) as well as several other genes such as *SUPPRESSOR OF OVEREXPRESSION OF CONSTANS 1* (*SOC1*), *LEAFY* (*LFY*), *SEPALATA* (*SEP*), and *FRUITFUL* (*FUL*), and triggers flowering. The natural *B*. *napus* is considered a long-day plant [1, 17], where the CO protein is not expected to accumulate at a normal rate under a short day condition; this would result in reduced activation of *FT* transcription and consequently delay flowering. In the present study, we detected a single QTL on C9 at 10 h photoperiod condition; this QTL found to exert an additive effect as well as show significant interaction with the C1 QTL, which could be detected in all environments and affect days to flowering apparently through the autonomous pathway. This indicates that the genomic region of C9 might carry a gene affecting flowering time where the allele introgressed from *B*. *oleracea* var. *alboglabra* is capable of enhancing flowering at 10 h photoperiod condition either directly or in interaction with the gene located on C1. Given the fact that the *B*. *napus* parent RIL-144 flowered significantly earlier than Hi-Q at 10 h photoperiod condition and none of the DH lines flowered significantly earlier than RIL-144 under this condition, the earliness in this parent and in the DH population apparently resulted from additive effect of the QTL alleles of C1 and C9 and their interaction. Interaction between these two main-effect flowering time QTL was detected only under this short-day (10 h photoperiod) condition.

Zaman *et al*. [42] studied the genetic control of photosensitivity by use of a population derived from crossing of photoinsensitive and photosensitive *B*. *napus* lines and found the evidence of multiple gene control of this trait with possible involvement of a locus with large effect. Cai *et al*. [21] detected four photosensitive QTL on A3, A10, C4 and C8 in a spring *B*. *napus* population. Robert *et al*. [19] isolated and characterized four *B*. *napus* homologues of the *Arabidopsis CO* gene and mapped these on A10 and C9. By using a DH population developed from crossing of two spring type *B*. *napus* parents, where one parent contained genome contents introgressed from an early-flowering *B*. *rapa*, Lou *et al*. [22] detected four flowering time QTL on A6, A7, C8 and C9 in three environments and one QTL on C2 in one environment. Of these two C-genome QTL, the C9 QTL was found to be involved in photoperiod-responsive flowering while the C8 QTL was found not to be influenced by environment and might be involved in the control flowering through the autonomous pathway. By working with a population derived from *B*. *oleracea* var. *alboglabra* × var. *italica*, requiring no vernalization for flowering, Bohuon *et al*. [43] detected six QTL on the linkage groups O2 (C2), O3 (C3), O5 (C5) and O9 (C9) affecting days to flowering. They also provided evidence that the QTL region of O9 show collinearity with a region of *A*. *thaliana* chromosome 5 carrying a *CO* photoperiod-responsive gene. Axelsson *et al*. [20] identified two QTL regions of C3 and C9 of *B*. *oleracea* carrying the *CO* gene. Okazaki *et al*. [44] also identified a QTL on O9 and found evidence for the presence of a *CO* gene in this genomic region. Thus, there is evidence that the *Brassica* C genome chromosome carry a gene that can affect flowering time through the photoperiod pathway. Data presented in this paper demonstrate that the chromosome C9 carry a photoperiod-responsive gene; we introgressed an allelic variation of this gene from *B*. *oleracea* var. *alboglabra* into spring *B*. *napus* canola [23]. Most of the above-mentioned researchers used RFLP, SRAP and CAPS markers while we used the SSR markers; therefore, no direct comparison between the markers from the C9 QTL region(s) of the present and previous studies could be made. BLASTn search identified several flowering time genes on the chromosomes C1 and C9; the QTL region of C1 bracketed by the markers sN11912 and sN0842 found to carry two putative flowering genes. Whether these genes are involved in flowering time variation in this DH population needs to be investigated.

The genes involved in autonomous pathway are not influenced by photoperiod or temperature. In the present study, by growing the mapping population under different photoperiod and temperature conditions, we detected a QTL on C1 which has not been affected by these two environmental factors. Previously, we [31] also detected this QTL in nine field trials, conducted over five years in Alberta (about 50 km radius of Edmonton), under similar day length condition. Different researchers, such as Rae *et al*. [45], Chen *et al*. [46] and Raman *et al*. [13, 47, 48], detected flowering time QTL on this chromosome; however, this QTL could not be detected consistently in their studies–apparently due to sensitivity of the QTL to the environment. Comparison of the genomic region of the QTL detected by Raman *et al*. [13, 47, 48] showed that the C1 QTL detected by this research group is located at a different position as compared to the QTL detected in this study. Thus, the results from this study, as well as our previous study conducted in eight field trials over five years [31], provide substantial evidence that the C1 QTL region we detected (Fig 1) carry a gene which control flowering time without being influenced by temperature, photoperiod and field trials conditions. Whether this QTL would be affected by other environmental factors, such as light intensity, drought, and nutrient availability, needs further investigation. The C1 QTL allele resulting earliness of flowering apparently not present in natural *B*. *napus* canola population–we introgressed this novel allele from *B*. *oleracea*.

To date, several genes, such as *LUMINIDEPENDENS (LD)*, *FCA*, *FY*, *FPA*, *FD*, *FVE*, *FLK*, and *REF6*, are reported to be involved in autonomous pathway of flowering [41]. BLASTn search of the C1 QTL region identified two putative genes to be associated with flowering time–the Gene ID 106374859 located at physical position of 32 873 610 nt (start position) to 32 875 196 nt (end position) encoding “*zinc finger protein CONSTANS-LIKE 12-like*” and the Gene ID 106376922 located at physical position of 33 189 464 nt (start position) and 33 192 085 nt (end position) encoding “*protein EARLY FLOWERING 3-like*” [31]; however, functional property of these genes need to be studied to determine whether any of these two genes is involved in autonomous pathway. Schiessl *et al*. [49] identified three flowering time genes on C1, viz. *FD*, *TERMINAL FLOWER 1* (*TFL1*) and *AGAMOUS-LIKE 24* (*AGL24*) of which *FD* is known to be involved in autonomous pathway [37, 41]. The *FD* expresses in meristem and activates the floral meristem identity genes through interaction with *FT* protein and thus initiate flowering; however, the genomic location of the three genes reported by Schiessl *et al*. [49] cannot be determined from their report. Therefore, presence of either of these genes, including a *FD* gene, in the QTL region of C1 identified in the present study cannot be ruled out. On the other hand, Scortecci *et al*. [50] reported that a mutation in *FLOWERING LOCUS M* (*FLM*) results in early flowering in *Arabidopsis thaliana* under both short and long day conditions, and heterozygous condition of this gene show intermediate phenotype; this gene show similarity with *FLC* and *AGL31*. Given the fact that the Brassica genomes evolved from an ancestral genome, similar to *Arabidopsis* [51], it would not be unlikely that the early flowering line RIL-144 carry a *flm* gene on C1. Significant interaction of the C1 QTL, which has not been influenced by photoperiod and temperature, with the C9 QTL, which could only be detected under a short day condition, suggests that interaction between the major genes involved in different pathways can occur. Scortecci *et al*. [52] reported that interaction of *flm* and *co* mutants result earliness of flowering in *A*. *thaliana*; this indicates that the C1 QTL might carry a *FLM* gene. There are also evidences that the *FLC* is involved in the autonomous pathway of flowering [53, 54]. Whether any of the two genes that we identified on C1 through BLASTn search is a *FD*, *FLM*, *FLC*, *AGL* or *TFL* needs to be determined.

In case of the vernalization pathway, *FLOWERING LOCUS C* (*FLC*) is the key regulator gene which represses the expression of the floral integrator genes *FT*, *FD* and *SOC1* and result failure of flowering. However, *FLC* is down-regulated by vernalization or low temperature and enable promotion of flowering by the floral integrator genes. The *FLC* is activated by its upstream regulator *FRIGIDA* (*FRI*) gene; these two genes (*FRI* and *FLC*) are, therefore, the major determinants for natural variation of flowering in response to vernalization or low temperature. Beside the genes involved in vernalization pathway, the gene *TERMINAL FLOWER 1* (*TFL1*) is involved in regulation of flowering time in response to ambient temperature [55] and is also an important integrator of different flowering pathways [56], and, therefore, important for flowering time variation in spring growth habit plants. However, in the present study, we were not able to detect any additional QTL in the experiment where night temperature was lowered to 8°C.

In the present study, we also identified additional genomic regions of the chromosomes C2, C8 affecting flowering time; however, none of these QTL could be detected in all experiments. Therefore, it is probable that these genomic regions carry genes which are sensitive to environment. Lou *et al*. [22] detected a QTL on C2 by growing a spring *B*. *napus* DH population in a winter-crop area in China; however, this QTL could not be detected while the population was grown in a spring-crop area. By use of a spring growth habit DH population, derived from crossing of a late and an early flowering lines, and testing in spring and winter growth environments, Liu *et al*. [57] detected 22 QTL from eight chromosomes of *B*. *napus* including two C genome chromosomes C2 and C8; the C2 QTL was detected in all five environments while the C8 QTL was detected only in the winter environment. Chen *et al*. [46] also detected an environment-sensitive QTL on C8 by using a spring *B*. *napus* population. Zou *et al*. [58] reported that the C genome chromosomes C2, C3, C4, C6, C7 and C8 of *B*. *carinata* carry QTL affecting flowering time. Thus, there are evidences to support that the chromosomes C2 and C8 carry QTL which can affect flowering time variation in *Brassica*.

In conclusion, by use of a spring *B*. *napus* mapping population carrying the genome content of *B*. *oleracea* and studying this population under different photoperiod and temperature conditions, we identified six QTL from the linkage groups C1, C2, C8 and C9 affecting flowering time. Among these, the C1 QTL affected flowering time without being influenced by photoperiod and temperature while the C9 QTL could be detected only under a short photoperiod condition; these two QTL also showed significant interaction in the control of this trait. Thus, we provide substantial evidence that the late flowering species *B*. *oleracea* carry flowering time alleles which are not sensitive to photoperiod and temperature as well as alleles which can induce flowering under a short day condition. We also provide evidence of the value of the gene pools of the allied species not only for the improvement of earliness in *B*. *napus* canola, but also for unveiling the genomic regions of this amphidiploid species affecting a trait. The QTL regions and molecular markers identified in this study can potentially be used in marker-assisted selection for the improvement of the earliness of flowering, as well as for fine mapping and dissection of the genomic regions, especially the QTL region of C1 and C9, for identification of candidate genes involved in the control of flowering time in the C genome.

## Supporting information

S1 File Analysis of variance, spearman’s correlation coefficient among the replications, descriptive statistics, and coefficient of correlation between the growth chamber experiments for days to flowering of DH lines of Hi-Q × RIL-144 of *Brassica napus*. (DOC)

S2 File List of the genes on the chromosomes C1 and C9 of *Brassica napus*. (XLSX)
