# Supplementary material for: Molecular mapping of QTL alleles of Brassica oleracea affecting days to flowering and photosensitivity in spring Brassica napus
Source: PLoS One. 2018 Jan 10;13(1):e0189723. doi: 10.1371/journal.pone.0189723 (PMC5761838; doi:10.1371/journal.pone.0189723)
Supplement: S1 File — (DOC) [file pone.0189723.s001.doc]

**Supporting information: S1 File**

**Table A. Analysis of variance for days to flowering of the doubled haploid lines derived from Hi-Q × RIL-144 cross of *Brassica napus* tested under three photoperiods (10, 14 and 18 h) and 18 ºC constant temperature condition**.

| **Effect** | **Num DF** | **F Value** | **Pr > F** |
| --- | --- | --- | --- |
| Genotype | 93 | 3.8 | <.0001 |
| Photoperiod | 2 | 3710.8 | <.0001 |
| Genotype (Photoperiod)^a^ | 186 | 1.9 | <.0001 |

^a^ A mixed model where photoperiod is nested.

**Table B. Spearman’s correlation coefficients between the replications (repetition of the experiment in growth chamber) of the experiments conducted under different photoperiod and temperature conditions for days to flowering of the doubled haploid lines of Hi-Q × RIL-144 cross of *Brassica napus*.**

1. 10 h photoperid and 18 ᴼC constant temperature

|  | Rep. 2 |
| --- | --- |
| Rep. 1 | 0.480*** (86)^a^ |
| ***p<0.001; ^a^in brackets, *df* |  |

1. 14 h photoperid and 18 ᴼC constant temperature

|  | Rep. 2 | Rep. 3 |
| --- | --- | --- |
| Rep. 1 | 0.637*** (89)^a^ | 0.614*** (88) |
| Rep. 2 |  | 0.631*** (85) |
| *** p<0.001; ^a^in brackets, *df* | | |

1. 18 h photoperid and 18 ᴼC constant temperature

|  | Rep. 2 | Rep. 3 |
| --- | --- | --- |
| Rep. 1 | 0.496*** (90)^a^ | 0.498*** (90) |
| Rep. 2 |  | 0.920*** (90) |
| *** p<0.001; ^a^In brackets, *df*   1. 16 h photoperid and 18/8 ᴼC (day/night) temperature  |  | Rep. 2 | Rep. 3 | | --- | --- | --- | | Rep. 1 | 0.541*** (88)^a^ | 0.541*** (92) | | Rep. 2 |  | 0.760*** (88) | | *** p<0.001; ^a^in brackets, *df* | | | | | |

(v) 16 h photoperid and 20 ᴼC constant temperature

|  | Rep. 2 | Rep. 3 | Rep. 4 | Rep. 5 |
| --- | --- | --- | --- | --- |
| Rep. 1 | 0.744*** (76) | 0.498*** (76) | 0.575*** (76) | 0.562*** (73) |
| Rep. 2 |  | 0.552*** (74) | 0.615*** (74) | 0.554*** (72) |
| Rep. 3 |  |  | 0.785*** (90) | 0.751*** (86) |
| Rep. 4 |  |  |  | 0.746*** (88) |
| *** p<0.001; ^a^In brackets, *df* | | | | |

**Table C. Descriptive statistics based on LSmeans of the replicated experiments for days to flowering of the parents and doubled haploid lines of Hi-Q × RIL-144 cross of *Brassica napus* grown under different photoperiod and temperature conditions. The early flowering *B. napus* cv. Peace is included as check.**

| Population^1^ | Mean ± SD | Range |
| --- | --- | --- |
| 1. *10 h photoperid and 18 ᴼC constant temperature* | | |
| DH lines | 74.6 ± 9.9 | 56 - 97 |
| Hi-Q | 92.2 ± 7.6 | 85 -100 |
| RIL-144 | 56.9 ± 7.6 | 53 - 59 |
| F_1_ (Hi-Q × RIL-144) | 80.7 ± 7.6 | 60 - 90 |
| Peace | 73.0 ± 7.6 | 59 - 75 |

1. *14 h photoperid and 18 ᴼ*C constant temperature

| DH lines | 31.9 ± 1.7 | 29 - 37 |
| --- | --- | --- |
| Hi-Q | 33.9 ± 1.1 | 31 - 37 |
| RIL-144 | 29.9 ± 1.1 | 27 - 34 |
| F_1_ (Hi-Q × RIL-144) | 30.5 ± 1.0 | 26 - 34 |
| Peace | 31.0 ± 1.1 | 27 - 34 |

1. *18 h photoperid and 18 ᴼ*C constant temperature

| DH lines | 33.0 ± 1.9 | 30 - 41 |
| --- | --- | --- |
| Hi-Q | 35.3 ± 1.0 | 30 - 44 |
| RIL-144 | 32.3 ± 1.0 | 27 - 38 |
| F_1_ (Hi-Q × RIL-144) | 32.3 ± 1.0 | 28 - 40 |
| Peace | 32.9 ± 1.0 | 27 - 40 |

1. *16 h photoperid and 18/8 ᴼ*C (day/night) temperature

| DH lines | 42.0 ± 3.4 | 37 - 50 |
| --- | --- | --- |
| Hi-Q | 46.5 ± 2.7 | 44 - 49 |
| RIL-144 | 37.6 ± 2.7 | 31 - 41 |
| F_1_ (Hi-Q × RIL-144) | 40.2 ± 2.7 | 35 - 46 |
| Peace | 37.4 ± 3.8 | 29 - 42 |

1. *16 h photoperid and 20 ᴼ*C constant temperature

| DH lines | 34.8 ± 2.9 | 32 - 45 |
| --- | --- | --- |
| Hi-Q | 36.1 ± 0.4 | 34 - 39 |
| RIL-144 | 33.3 ± 0.4 | 32 - 35 |
| F_1_ (Hi-Q × RIL-144) | 33.3 ± 0.9 | 31 - 36 |
| Peace | 36.6 ± 2.1 | 32 - 40 |

^1^Number DH line tested in all experiments was 94 except 16 h photoperiod and 20 ᴼC constant temperature where 97 lines tested.

## Table D. Coefficient of correlations for days to flowering between the growth chamber experiments conducted under different photoperiod and temperature conditions with a set of doubled haploid lines derived from Hi-Q × RIL-144 cross of *Brassica napus*. LSmeans data were used to calculate the coefficient of correlation values.

|  | 14h 18 ᴼC | 18h 18 ᴼC | 16h 18/8 ᴼC | 16h 20 ᴼC |
| --- | --- | --- | --- | --- |
| 10h 18 ᴼC^a^ | 0.578 | 0.481 | 0.475 | 0.561 |
| 14h 18 ᴼC |  | 0.744 | 0.668 | 0.673 |
| 18h 18 ᴼC |  |  | 0.619 | 0.624 |
| 16h 18/8 ᴼC |  |  |  | 0.607 |

^a^Growth chamber conditions: 10h 18 ᴼC = 10 h photoperiod and 18 ᴼC constant temperature; 14h 18 ᴼC = 14 h photoperiod and18 ᴼC constant temperature; 18h 18 ᴼC: = 18 h photoperiod and 18 ᴼC constant temperature; 16h 20 ᴼC = 16 h photoperiod and 20 ᴼC constant temperature; 16h 18/8 ᴼC = 16 h photoperiod and 18/8 ᴼC (day/night) temperature. Note: Probablty of the coefficients of correlation is highly significan in all cases (*p* ≤ 0001). Degree of freedom (df) in all cases is 92 except 10h 18 ᴼC vs. 18h 18 ᴼC and 10h 18 ᴼC vs. 16h 20 ᴼC, where *df* were 93 and 94 respectively.
